# Plant Genome Engineering for Targeted Improvement of Crop Traits

**DOI:** 10.3389/fpls.2019.00114

**Published:** 2019-02-12

**Authors:** Khalid E. M. Sedeek, Ahmed Mahas, Magdy Mahfouz

**Affiliations:** Laboratory for Genome Engineering and Synthetic Biology, Division of Biological Sciences, King Abdullah University of Science and Technology, Thuwal, Saudi Arabia

**Keywords:** CRISPR/Cas systems, genome editing, genome engineering, crop improvement, climate change, food security, synthetic biology

## Abstract

To improve food security, plant biology research aims to improve crop yield and tolerance to biotic and abiotic stress, as well as increasing the nutrient contents of food. Conventional breeding systems have allowed breeders to produce improved varieties of many crops; for example, hybrid grain crops show dramatic improvements in yield. However, many challenges remain and emerging technologies have the potential to address many of these challenges. For example, site-specific nucleases such as TALENs and CRISPR/Cas systems, which enable high-efficiency genome engineering across eukaryotic species, have revolutionized biological research and its applications in crop plants. These nucleases have been used in diverse plant species to generate a wide variety of site-specific genome modifications through strategies that include targeted mutagenesis and editing for various agricultural biotechnology applications. Moreover, CRISPR/Cas genome-wide screens make it possible to discover novel traits, expand the range of traits, and accelerate trait development in target crops that are key for food security. Here, we discuss the development and use of various site-specific nuclease systems for different plant genome-engineering applications. We highlight the existing opportunities to harness these technologies for targeted improvement of traits to enhance crop productivity and resilience to climate change. These cutting-edge genome-editing technologies are thus poised to reshape the future of agriculture and food security.

## Food Security: Addressing Old Challenges and Emerging Threats

To sustain life, food must provide an adequate supply of calories and nutrients. Food insecurity, the lack of access to an adequate food supply, threatens millions of people worldwide with malnutrition. Moreover, the problem is getting worse; the global population is growing rapidly and is expected to reach 8.3 billion by 2030 (United Nations, Department of Economic and Social Affairs, Population Division, 2017). As a result, the demand for food, animal feed, and fuel will increase (Sundström et al., 2014). Challenges to food security, such as increasing population, have been joined by new threats such as increases in abiotic stresses due to climate change, decreases in arable land due to desertification, salinization, and human use, and emerging diseases. To enhance food security for future generations, the world must double the current crop production rate in spite of the predicted threats, including climate change (Godfray et al., 2010; Jones et al., 2014). Plant breeders have harnessed natural and artificial mutations, as well as important tactics such as breeding for hybrid vigor, to address food insecurity. However, additional work will be required to meet current and emerging challenges.

To improve crop yield, current approaches aim to increase the amount of food produced per unit of area cultivated, and to prevent crop failures. To increase yield per area in grain crops such as rice, breeders have targeted traits that increase the number of grains produced per plant, the number of plants that can be cultivated per unit area, and the size of each grain. Many of these traits involve manipulation of plant architecture through balancing meristem activity and hormone signaling. To prevent crop failures and thus improve yield stability, breeders have targeted traits that help crops tolerate stresses. For abiotic stress, researchers have targeted tolerance to heat, cold, high light, high salt, heavy metals, and other stresses. For biotic stresses, which have become an increasing problem as globalization and weather accelerate the spread of pathogens, researchers have identified loci conferring resistance to various viral, bacterial, and fungal pathogens, as well as loci affecting interactions with animal and plant pathogens, including nematodes and parasitic plants such as Striga (Butt et al., 2018). The challenge in disease resistance is twofold, identifying the essential loci to introduce, and introducing the key resistance loci into elite varieties in a timely manner. Moreover, balancing the energy requirements for resistance and growth to minimize yield penalties remains difficult.

To increase the nutrition of crops, current approaches aim to provide diverse and balanced diets with adequate levels of vitamins and minerals that enhance human health. Recent developments in crop biotechnology make it possible to manipulate the key enzymes in certain metabolic pathways, thereby enhancing the contents of key nutrients such as vitamins and iron, and reducing the contents of unfavorable compounds such as phytic acids and acrylamide-forming amino acids. Several biofortified crops such as rice, maize, and wheat have been produced to solve the problem of nutrition deficiencies (Ye et al., 2000; Gil-Humanes et al., 2014; Mugode et al., 2014). A well-known example is Golden Rice, which is genetically engineered to produce a significant level of β-carotene to help people at risk of vitamin A deficiency (Ye et al., 2000).

## A Historical Perspective on Plant Genome Engineering

Nature has been altering genomes for thousands of years, with natural selection enabling plants with certain genomic variants to survive. Moreover, humans have been using artificial selection to domesticate crops for more than 10,000 years. This process produced modern corn from its wild ancestor teosinte, among many other examples. Indeed, all crops grown today have undergone extensive genetic changes. Genetic changes or variations are key to crop improvement, but our ancestors had to make do with naturally occurring mutations. In the twentieth century, once it was recognized that DNA and genes shape all life, it became clear that altering DNA sequences induces phenotypic variations. Therefore, researchers developed and tested reagents, including radiation and chemical mutagens, to induce DNA mutations and have examined the resulting phenotypic variations (Shu et al., 2012). This mutation breeding concept was established in the 1940s and yielded noteworthy successes, such as the wheat varieties with significantly improved yields that were key to the Green Revolution of the 1970s.

A major advance in genetic modification was made with the discovery that *Agrobacterium tumefaciens* (Agrobacterium), the bacterium that causes crown gall disease, is a natural genetic engineer that introduces a piece of its own DNA into the genome of a plant it infects, potentially carrying along a DNA sequence provided by a researcher (Nester, 2014). This bacterium injects a so-called tumor-inducing (Ti) plasmid into the plant cell, where it integrates into the genome (Yadav et al., 1982). Engineering of Ti-plasmid-derived “binary vectors” that can replicate in *Escherichia coli* as well as in Agrobacterium, and still integrate into plant genomes, provided the basis for plant biotechnology. Using these tools, it is possible to incorporate into a plant genome even genes from distantly related organisms, in a process called transgenesis; if the genes come from related plant species, this process is called cisgenesis (Schubert and Williams, 2006). However, this approach has many drawbacks, including the random nature of the gene insertion, the possibility of disrupting functional genes, public concerns over genetically modified organisms (GMOs), and the failure to make use of the native genetic repertoire of the plant. There was therefore a pressing need for techniques to precisely change DNA sequences at the single-base level. Such technologies for adding, deleting, and editing existing DNA sequences to develop traits of interest are essential to crop bioengineering for various purposes, including improving crop performance to withstand the hotter and drier environments expected to arise under climate change.

**FIGURE 1:**
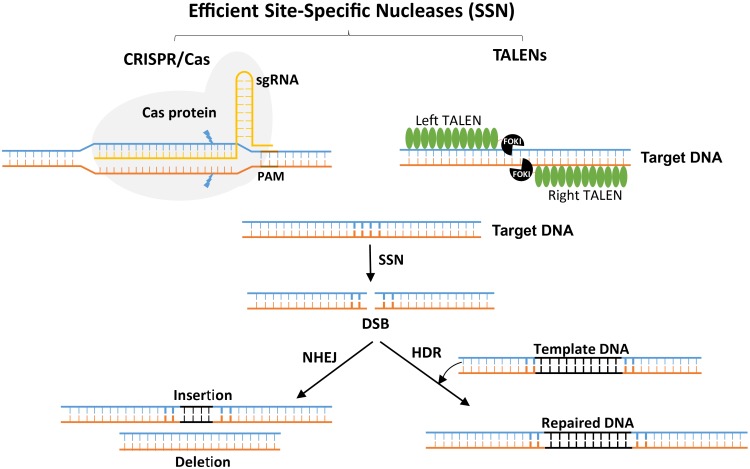
Site-specific nuclease-induced genome editing. The double-stranded breaks (DSBs) introduced by CRISPR/Cas or TALEN complexes stimulates the endogenous DNA repair machineries, non-homologous end joining (NHEJ) or homology-directed repair (HDR). The NHEJ machinery repairs the DNA imperfectly and introduces frameshifts by insertion or deletion leading to loss-of-function mutations. However, the HDR pathway precisely inserts a piece of DNA (from an exogenous template DNA with enough similarity to the DSB flanking sequence) by homologous recombination, which is useful for the introduction of specific point mutations or a new gene sequence.

In the 1980s, Mario Capecchi first established gene-targeting technology, along with the concept of harnessing double-strand breaks (DSBs) for genome editing (Capecchi, 1980). A later development was the ability to engineer genomes by generating site-specific DSBs (Jasin, 1996). After DSBs are generated, the cell’s own repair machinery can be harvested to dictate the genetic outcome through the imprecise repair process of non-homologous end joining (NHEJ) or the precise repair process of homology-directed repair (HDR) (Trevino and Zhang, 2014; Baltes and Voytas, 2015; Bortesi and Fischer, 2015; Schaart et al., 2016) (Figure 1). For example, NHEJ can cause insertion or deletion of a few bases and thus create functional knockouts of genes (Gorbunova and Levy, 1997; Charbonnel et al., 2011; Lloyd et al., 2012). By generating more than one DSB, it becomes possible to produce even more types of changes, including chromosomal deletions, gene inversions and, with DSBs on two different chromosomes, chromosomal translocations (Morgan et al., 1998; Ferguson and Alt, 2001). In contrast to NHEJ, HDR produces a precise repair and enables the sequence to be rewritten in a user-defined manner (Puchta et al., 1996; Puchta, 2005) (Figure 1). HDR can be used for genome editing and precise modification of the genome with various types of repair templates, ranging from short oligonucleotides to those a few hundred base pairs in length up to full genes with homologous ends or arms flanking the DSB site (Song and Stieger, 2017; Boel et al., 2018).

Generating DSBs allows many possible mechanisms of genome editing to be accomplished by harnessing the cell’s repair machinery. The big question was how to generate a site-specific DSB. Proteins that can be engineered and reprogrammed to bind and cleave DNA do not exist in nature. However, it is possible to program a DNA-binding domain to bind to any user-defined site-specific sequence. This domain can be fused with another domain that can cleave the DNA specifically where it binds. These bimodular fusion proteins are the key to precise genome engineering because they can be programmed to bind to any user-selected sequence and generate a DSB. Such programmable site-specific binding proteins can carry other functional domains capable of effecting other genetic and genomic changes, including transcriptional regulation, epigenetic regulation, and even base editing without generation of DSBs (Komor et al., 2016; Puchta, 2016). The genome-engineering toolbox has three major platforms: zinc-finger nucleases (ZFNs), transcription activator-like effector nucleases (TALENs), and CRISPR/Cas systems. ZFNs and TALENs are protein-based and require protein engineering for every user-defined sequence. However, CRISPR/Cas is an RNA-guided system and can be easily engineered to bind to the DNA target (Belhaj et al., 2013; Osakabe and Osakabe, 2015; Quétier, 2016). TALENs and CRISPR/Cas9 have been used to produce many key agricultural innovations; therefore, we focus on these systems below.

## Talen-Based Systems

In nature, the phytopathogen *Xanthomonas oryzae* (Xanthomonas) produces TAL effectors (TALEs), which enter the plant cell nucleus and reprogram the transcription machinery to benefit the pathogen (Doyle et al., 2013). They function as eukaryotic transcription factors by binding to the promoter region and activating gene expression. TALEs have unique structural features, including a central DNA-binding repeat that dictates DNA binding specificity through a one repeat to one base pair binding correspondence (Deng et al., 2012; Doyle et al., 2013). By engineering the number and type of these repeats, TALEs can be engineered to bind any DNA sequence (Li L. et al., 2012). Fusion of a TALE with a nuclease produces an enzyme that can generate site-specific DSBs *in vitro* and *in vivo* (Christian et al., 2010; Mahfouz et al., 2011).

The structural basis of TALE-DNA binding is amino acid 12 of the TALE repeat sequence, known as the repeat variable di-residue (RVD), which facilitates and stabilizes the contact, and amino acid 13, which confers binding specificity (Boch et al., 2009; Deng et al., 2012). The DNA-binding specificities of TALEs allows them to serve as DNA-binding modules for building synthetic transcriptional and epigenetic regulators. Several engineering platforms have been developed for TALEs. Moreover, researcher have interrogated genomes from microbes other than Xanthomonas and determined that another bacterium, *Ralstonia solanacearum* (Ralstonia), possesses Ralstonia TALE-like proteins (RTLs) with similar structure but completely different repeats, along with enriched numbers of the RVDs that determine repeat specificity (Bogdanove et al., 2010).

Deciphering the code of RTL binding to DNA revealed that these RVDs provided a rich resource for TALEN-based engineering (Li L. et al., 2013). For example, the canonical TAL-binding RVD code (described using the single-letter amino acid code) is that the RVD HD binds to cytosine, NG binds to thymine, NN binds to adenine or guanine, and NS binds to any nucleotide. For RTLs, the DNA-binding code includes ND binding to cytosine, SH binding to guanine, NT binding to adenine, and HN binding to adenine or guanine, among others. These added binding specificities have provided diverse options and opportunities for TAL-based engineering (Li L. et al., 2013). Nonetheless, the requirement for engineering a specific protein for every target and the need for two TAL monomers to simultaneously bind the DNA strands makes TALEN-based genetic engineering time consuming and resource intensive (Joung and Sander, 2013; Nemudryi et al., 2014). Despite these challenges, many companies have chosen TALEN-mediated gene editing for its high precision and clear intellectual property landscape.

## CRISPR/Cas Systems

In nature, bacterial and archaeal species fend off invading phages and foreign genetic elements through the use of clustered regularly interspaced palindromic repeats (CRISPR)/CRISPR-associated protein (Cas) adaptive immune systems. About 40% of bacteria and most archaea have with several CRISPR/Cas systems capable of targeting DNA, RNA, or both for degradation, thereby defending themselves against foreign genetic elements (Jansen et al., 2002; Sorek et al., 2008). When a phage infects a bacterium equipped with CRISPR, the bacterium acquires pieces of the phage DNA within the CRISPR array in what is called the adaptation phase. Acquisitions are ordered with most recent one closest to the leader sequence, which functions as a promoter. The CRISPR array is transcribed and generates mature RNAs (known as crRNAs) in the biogenesis phase. Cas9 uses these crRNAs as guides to target the phage genome during future invasions and thereby provide immunity to the bacterial cell, marking the interference or immunity phase (Barrangou et al., 2007; Rath et al., 2015). Cas9 usually cleaves a DNA region that is 3–4 nucleotides upstream of a three-nucleotide protospacer-adjacent motif (PAM), which is not found in the bacterial genome, thus allowing this adaptive immune system to specifically target invading phages (Jinek et al., 2012).

CRISPR systems are classified into two main groups, classes I and II (Makarova et al., 2011, 2015). In class I systems (subdivided into types I, III, and IV), the interference complex is a multicomponent system composed of multiple effectors. In class II systems (types II, V, and VI), the interference complex is a single-component system and the interference complex comprises a single effector guided by the crRNA (Makarova et al., 2011, 2015; Shmakov et al., 2017). The CRISPR/Cas9 system, which belongs to class II, is a two-component system composed of Cas9 and a single guide RNA (sgRNA) molecule. Recently, other class II systems have been discovered, including some based on the Cas12a enzyme (previously known as Cpf1), which generates DSBs with staggered ends and has a T-rich PAM, thereby enriching the options available for genetic engineering in repetitive T-rich genomic regions across eukaryotic species (Zetsche et al., 2015). Also in class II are the type VI systems, based on the enzyme Cas13a, which is capable of targeting the RNA of viral species, thereby providing a very effective machinery for RNA interference in both prokaryotic and eukaryotic species (Abudayyeh et al., 2016).

### High-Efficiency Plant Genome Engineering Using CRISPR/Cas

**FIGURE 2:**
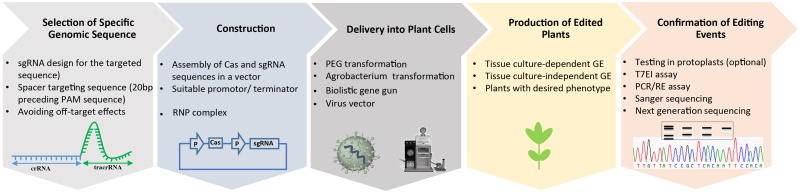
Simplified workflow for CRISPR/Cas9-mediated plant genome editing. The production of edited plants with a desired phenotype starts from the design of a sgRNA for a specific target sequence and cloning of the sequence to express the sgRNA into a binary vector containing the Cas DNA sequence. Then the delivery of CRISPR/Cas materials into the plant cell, followed by assays to confirm the presence of the edited events, and regeneration of whole plants.

For high-efficiency genome engineering in any eukaryotic cell, it is necessary to ensure that delivery of the genome-engineering reagents to the appropriate species be feasible and that editing of the target genome is both highly specific and efficient (Figure 2). Therefore, reagent delivery and editing specificity are key research areas for developing high-efficiency genome-engineering technologies. For plants, a current major focus is on developing delivery platforms for genome-engineering reagents, preferably for delivery into germline cells to bypass the need for tissue culture and regeneration after editing (Forner et al., 2015; Mao et al., 2016). Delivery platforms include bacterial and viral vectors, and physical delivery into different types of cells. Specificity research involves the identification of Cas9 variants that are inherently more specific than current enzymes and have optimized expression and sgRNA architectures, as well as the titration of sgRNA and Cas9 concentrations during the editing process. Editing research involves developing effective HDR technologies that provide ultimate control over the repair process and the genetic outcome, including the ability to generate gene fusions, targeted gene replacement and additions, and single-base substitutions (Ochiai, 2015; Vanden Bempt et al., 2016; Zhao et al., 2016). Efficient editing remains challenging in most eukaryotic cells, and several research efforts focused on improving gene editing are detailed below.

#### Efficient Delivery Vehicles for Genome-Engineering Reagents

Engineering of the CRISPR/Cas9 system currently means simply engineering the sgRNA molecule, which provides targeting specificity and can also include a template for HDR. Therefore, we sought to develop a system in which we could use a virus as the vehicle for sgRNA delivery into plants expressing Cas9 (Ali et al., 2015b). This approach involves the generation of a Cas9 overexpression line in a model plant species such as *Nicotiana benthamiana* or *Arabidopsis thaliana* and the subsequent delivery of sgRNAs via *Tobacco rattle mosaic virus* (TRV). After establishment of the TRV infection in the Cas9 overexpression line, we assayed for modification of the genomic target sequence. To further improve the efficiency of this approach and increase the recovery of seed progeny carrying the modification, we recently tested delivery with *Pea early browning virus* (PEBV), which is capable of infecting the germline (Ali et al., 2018a). When we compared the efficiencies of TRV and PEBV for targeted mutagenesis of somatic tissues, we found that PEBV is highly efficient (Ali et al., 2018a).

The viral delivery system provides two options: (1) tissue-culture-free genome editing, in which the CRISPR/Cas9 machinery is active in the germline, and (2) tissue-culture-dependent genome engineering. Some RNA viruses are capable of infecting germline cells, albeit at low frequency, and this would enable the recovery of progeny carrying the intended genomic modification, as discussed in more detail below (Ali et al., 2018a). We can also start with leaf tissue, where the efficiency of our genome-engineering system is good, and regenerate whole plants, which we can then genotype for the presence of the modification (Ali et al., 2015b, 2018a; Aman et al., 2018). Therefore, the advantages of viral systems include the potential to perform tissue-culture-free genome editing, high-efficiency targeted mutagenesis, and also the possibility to do functional genomics experiments using a sgRNA library constructed in the viral vector, as detailed below.

Among prokaryotic vectors, Agrobacterium is a natural genetic engineer because of its ability to transfer a piece of its genome, the transfer DNA (T-DNA), into the plant genome (Nester, 2014). This intriguing interkingdom DNA transfer is facilitated by the virulence (vir) proteins, which are encoded by the Ti plasmid and facilitate DNA nicking, processing, transfer, and integration into the plant genome (Hoekema et al., 1983). The T-DNA is transferred through the type IV secretion system, along with many bacterial proteins, and eventually enters the cell nucleus where it integrates randomly into the plant genome. Some of these virulence proteins make the trip from the bacterial cell into the plant cell regardless of whether T-DNA transfer occurs. One intriguing possibility would be to use some of those proteins to deliver ribonucleoproteins (RNPs) from the bacterium into the plant cell nucleus, as this could make it possible to produce the CRISPR/Cas9 machinery in bacteria and then deliver it intact into plant cells, allowing researchers to recover seed progeny carrying the desired gene edits without the need for classical tissue culture.

#### Germline Engineering via CRISPR/Cas9

Current plant genome-engineering efforts are primarily conducted through classical transformation and tissue culture, as with transgenesis approaches. This limits the application of CRISPR/Cas technologies in crop species, especially those that are recalcitrant to Agrobacterium transformation or to regeneration. There is thus a pressing need to develop technologies that do not rely on classical transformation and regeneration of transformed cells. The ideal target cell types for this approach are the germline cells, where delivery of CRISPR/Cas9 machinery in DNA or protein form can permanently change the genotype. RNP-mediated engineering of germline cells would be ideal given the regulatory hurdles associated with DNA-based editing and the need to produce plants that are free of foreign DNA.

As mentioned above, some viral systems can deliver sgRNAs to germ cells. Several other approaches can be used, including direct delivery of the reagents via Agrobacterium and isolation of the germline cells for polyethylene glycol (PEG)-mediated transfection (Mao et al., 2016). Other approaches using biolistic gene guns, electroporation, optoporation, magnetofection, or microinjection are appropriate to some germline cells, depending on the plant species and developmental stage (Mohanty et al., 2016). Select nanoparticles can be used to deliver genome-engineering reagents in RNP form to target cells (Cunningham et al., 2018). Improving delivery methods would accelerate and expand the applications of plant genome engineering.

#### Single-Cell Genome Engineering

Because the CRISPR/Cas machinery is easy to engineer and has robust activity in plant cells, making individual cells with engineered genomes is quite efficient. However, producing whole plants from these cells remains challenging. For example, regeneration is often genotype dependent, and in most cases the cultivars used in laboratory experiments are not the elite germplasm used in agriculture (Altpeter et al., 2016). Moreover, with transformation methods generally use selectable markers like antibiotic- or herbicide-resistance genes.

Efficient single-cell regeneration will be a major achievement in plant biotechnology, and research in this area is ongoing on multiple fronts. Recent efforts have been made to deliver CRISPR/Cas9 in RNP form into the protoplasts of lettuce and tobacco, with subsequent editing and regeneration from single protoplast cells (Woo et al., 2015; Kim et al., 2017; Lee et al., 2018). Regeneration from protoplasts is quite inefficient in most plant species, however, limiting the application of this technology and the ability to produce foreign-DNA-free edited plants.

Recently, morphogenic factors have been used to enhance regeneration frequency (Altpeter et al., 2016; Lowe et al., 2016). Other possible avenues of approach are applying transient expression of shoot-specifying transcription factors to protoplast single-cell transformation, identifying effective strategies to boost regeneration competence of edited cells, and/or comparing the germplasm of cells with high regeneration frequency with that of cells that are recalcitrant to regeneration, with the aim of identifying regeneration boosters. Any or all of these approaches may improve the ability to regenerate plants from single cell, a key requirement for harnessing the power of CRISPR/Cas for genome-engineering applications.

#### CRISPR-Mediated Genome-Wide Functional Genomics Screens

**FIGURE 3:**
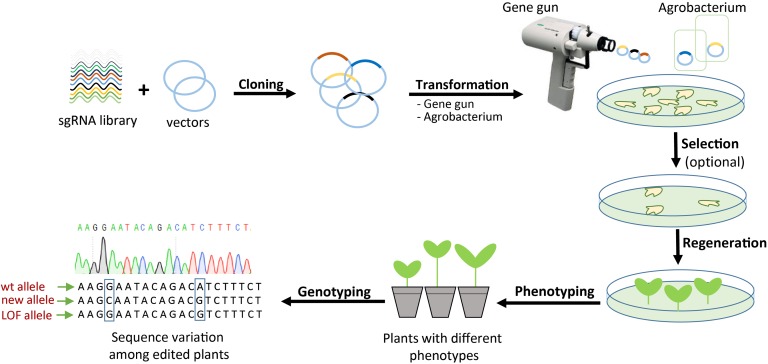
CRISPR-mediated genome-wide screening. Schematic illustration of the procedure for generating a wide variety of new plant traits by targeting one or several genes using a pool of sgRNAs. After generation of edited plants, deep phenotyping and genotyping screening are required to discover the interesting traits and their genetic background. LOF, loss of function.

CRISPR/Cas systems offer the ability to produce a variety of genetic and epigenetic modifications that could be instrumental to testing gene functions and regulation in the genomic context. It is feasible to develop CRISPR genome-wide screens as a gene discovery platform whereby sgRNAs are used to generate mutations or epigenetic changes in single or multiple genes (Sharma and Petsalaki, 2018). In the CRISPR GWS genome-wide screen system, it might be possible to construct sgRNA libraries that target the entire genome (Figure 3). The sgRNA libraries would then be cloned into binary vectors for plant transformation. Once the CRISPR/Cas machinery is expressed and seed progeny with modifications are recovered, preferably in one generation to ensure homozygosity of the modification, they can be subjected to screening to identify interesting phenotypes like resistance to abiotic or biotic stress factors, virus resistance, architecture, flowering, yield, and other traits of interest.

Once plants with the desired phenotype are identified, the causal genes can be easily identified by cloning and sequencing the sgRNA. The results can then be confirmed by expressing the wild-type allele of the candidate causal gene. In the past, this required time-consuming and laborious mapping efforts, which are quite challenging in many crops that are key for food security. Not only is CRISPR much more efficient at generating mutations than older (e.g., chemical) mutagens, but the nature of the mutations is different, meaning that adding CRISPR to the plant breeders’ toolbox will enrich plant populations and enhance the gene and trait discovery process.

These screens can be applied to either loss-of-function or gain-of-function platforms depending on which CRISPR/Cas system is used. Of particular utility would be the application of the CRISPR/Cas9 and CRISPR/Cas12a platforms, since they produce permanent changes in the genome and do not require the presence of the CRISPR/Cas9 machinery (Zetsche et al., 2015). An interesting modality would be the application of selective pressure during the expression of the CRISPR/Cas machinery, so as to force the generation of certain edits that could help plants resist those specific stress factors. Furthermore, for gene function analysis, researchers could use dCas9TF, Cas13a, or Cas13b along with base editors to transiently perturb key gene functions such as housekeeping and embryonic-lethal genes. These CRISPR systems are expected to allow very efficient gene and trait discovery not only in model species but in crop species as well, which will be crucial to improving crop yield and resilience under the unfavorable conditions of climate change.

#### CRISPR/Cas9 and TALEN Off-Target Activities

One major drawback to CRISPR/Cas9 systems is that they are prone to off-target activities (Zhang et al., 2015), owing to the ability of Cas9 to cut at other, unintended places in the genome in addition to the intended target sequence. This currently poses grave limitations on the use of CRISPR/Cas9 in gene therapy and genetic medicine. In contrast to CRISPR/Cas9, the TALEN system exhibit precision but delivery of TALENs is quite challenging.

Many approaches have been employed to reduce CRISPR/Cas9 off-target activity, including inducible systems to limit the availability window and concentration of Cas9, and different sgRNA architectures (Zhang et al., 2015; Cao et al., 2016). One strategy involves generating a chimeric fusion between a catalytically inactive Cas9 protein (dCas9) and the FokI catalytic domain. The inactive dCas9 is used as a targeting module to bring the FokI domain into close proximity and allow dimerization (Guilinger et al., 2014; Aouida et al., 2015), and the formation of homodimers with the right spacer sequence then allows the generation of DSBs. This dramatically increases the cutting specificity, because it requires 40 bp of unique sequence and a unique distance between the two monomers, thus limiting off-target activities (Yee, 2016). Several studies have indicated that off-target activities of Cas9 are not easily detected *in planta*, corroborating the general assumption that these off-target activities occur at very low levels in plants unlike a mammalian system where off-target activities is a serious problem (Ali et al., 2015b; Yee, 2016; Morgens et al., 2017).

### Targeted Improvement of Crop Traits

**FIGURE 4:**
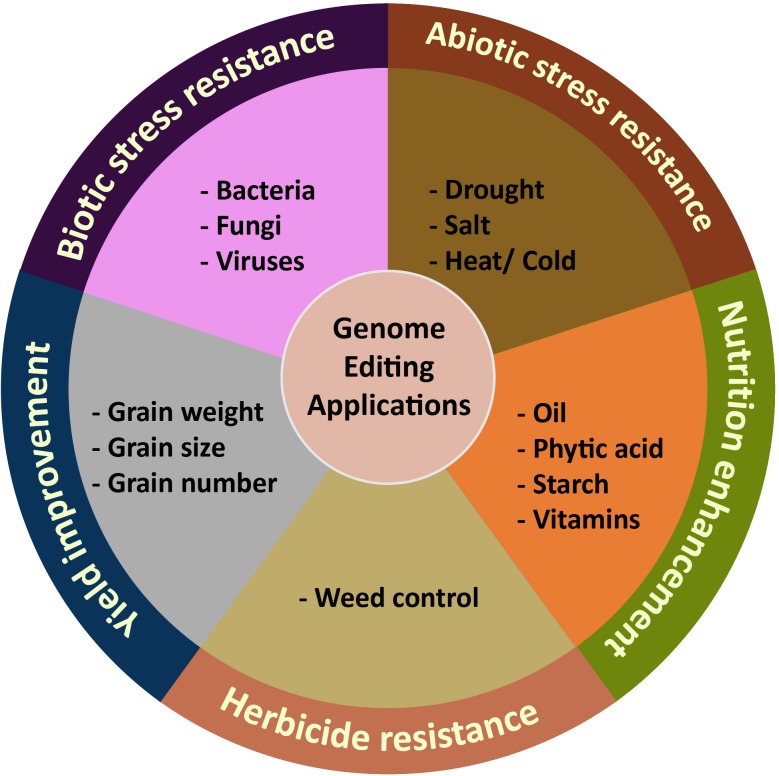
Application of plant genome editing for targeted trait improvement.

Although genome engineering is relatively new, the technology has been efficiently adapted to a wide range of crops as a means to improve yield, quality and nutritional value, herbicide resistance, and biotic and abiotic stress tolerance (Wang et al., 2016) (Table 1 and Figure 4). For identification of targets for genome editing, genetic studies have identified key yield-related loci and advanced sequencing technologies in crop species have produced key information on the sequence variation of trait-related genes. The identification of beneficial alleles that produce desirable phenotypes offers exciting possibilities for the use of genome engineering for accelerated and targeted trait improvement. Here, we provide highlights of key advances for improving crop traits using genome engineering and discuss the promise of these technologies for enhancing food security.

**Table 1:** Application of genome editing tools in different plant species to improve yield, biotic, and abiotic stress resistance, and nutritional quality.

Target trait	Plant species	Targeted sequence(s)	Results	Method	Reference
Yield	*Oryza sativa*	*GS3*, *Gn1a*	Grain size and number increase	CRISPR/Cas9	Shen et al., 2018a
	*Oryza sativa*	*GW2*, *GW5*, *TGW6*	Grain weight increase	CRISPR/Cas9	Xu et al., 2016
	*Oryza sativa*	*Gn1a*, *DEP1*, *GS3*	Grain size and number increase and dense, erect panicles	CRISPR/Cas9	Li M. et al., 2016
Virus resistance	*Arabidopsis thaliana*	*eIF(iso)4E*	Potyvirus resistance	CRISPR/Cas9	Pyott et al., 2016
	*Arabidopsis thaliana*	BSCTV genome	Beet severe curly top virus resistance	CRISPR/Cas9	Ji et al., 2015
	*Cucumis sativus*	*eIF4E^1^*	Cucumber vein yellowing virus, zucchini yellow mosaic virus, and papaya ring spot mosaic virus-W resistance	CRISPR/Cas9	Chandrasekaran et al., 2016
	*Nicotiana benthamiana*	BSCTV genome	Beet severe curly top virus resistance	CRISPR/Cas9	Ji et al., 2015
	*Nicotiana benthamiana*	TYLCV genome	Tomato yellow leaf curl virus resistance	CRISPR/Cas9	Ali et al., 2015a
	*Nicotiana benthamiana*	*AGO2*	Virus resistance	CRISPR/Cas9	Ludman et al., 2017
Fungus resistance	*Oryza sativa*	*OsERF922*	Rice blast resistance	CRISPR/Cas9	Wang et al., 2016
	*Solanum lycopersicum*	*SlMlo*	Powdery mildew resistance	CRISPR/Cas9	Nekrasov et al., 2017
	*Triticum aestivum*	*TaMLO-A1*	Powdery mildew resistance	CRISPR/Cas9 TALEN	Wang et al., 2014
Bacterial resistance	*Citrus sinensis* Osbeck	*CsLOB1*	Canker resistance	CRISPR/Cas9	Peng et al., 2017
	*Oryza sativa*	*OsSWEET13*	Bacterial blight resistance	CRISPR/Cas9	Zhou et al., 2015
	*Oryza sativa*	*Os11N3* (*OsSWEET14*)	Bacterial blight resistance	TALEN	Li T. et al., 2012
Drought tolerance	*Arabidopsis*	*mir169a*	Improved drought tolerance	CRISPR/Cas9	Zhao et al., 2016
	*Zea mays*	*ARGOS8*	Improved grain yield under field drought stress conditions	CRISPR/Cas9	Shi et al., 2017
Salt tolerance	*Oryza sativa*	*OsRAV2*	Salt stress tolerance	CRISPR/Cas9	Duan et al., 2016
Herbicide tolerance	*Linum usitatissimum*	*EPSPS*	Glyphosate tolerance	CRISPR/Cas9	Sauer et al., 2016
	*Nicotiana tabacum*	*MEL1*	Herbicide tolerance	ZFN	Cai et al., 2009
	*Nicotiana tabacum*	*ALS*	Resistance to imidazolinone and sulfonylurea herbicides	TALEN	Zhang et al., 2013
	*Oryza sativa*	*ALS*	Chlorsulfuron and bispyribac sodium tolerance	CRISPR/Cas9	Sun et al., 2016
	*Oryza sativa*	*EPSPS*	Glyphosate tolerance	CRISPR/Cas9	Li J. et al., 2016
	*Solanum tuberosum*	*ALS1*	Chlorsulfuron and bispyribac sodium tolerance	CRISPR/Cas9	Butler et al., 2016
	*Zea mays*	*IPK1*	Herbicide tolerance	ZFN	Shukla et al., 2009
Nutritional improvement	*Camelina sativa*	*FAD2*	Enhancement of seed oil	CRISPR/Cas9	Jiang et al., 2017
	*Oryza sativa*	*SBEI*, *SBEIIb*	High amylose content	CRISPR/Cas9	Sun et al., 2017
	*Oryza sativa*	*OsBADH2^2^*	Increased fragrance content	TALEN	Shan et al., 2015
	*Solanum tuberosum*	*GBSS*	High-amylopectin starch	CRISPR/Cas9	Andersson et al., 2017
	*Zea mays*	*ZmIPK*	Reduced phytic acid content	CRISPR/Cas9 TALEN	Liang et al., 2014


*^*1*^eIF4E, eukaryotic translation initiation factor 4E. ^*2*^BADH, betaine aldehyde dehydrogenase.*

#### Improving Yield

Yield is one of the most important traits for crop plants. It is a quantitative trait, controlled by several genes (Xing and Zhang, 2010; Bai et al., 2012), and considerable research has been conducted to identify the quantitative trait loci (QTLs) controlling yield in various crop plants (Bai et al., 2012; Jianru and Jiayang, 2014). Traditional breeding, the original method used to improve yield and develop plants able to survive in particular growth environments (Duvick, 1984), is a time-consuming process. Breeding relies on generating various combinations of QTLs and selecting the most promising ones for further breeding (Xufeng et al., 2012; Zuo and Li, 2014; Shen et al., 2018b). In addition, the introgression of QTLs between different varieties is not always easy, especially with closely linked loci.

Genome editing provides a promising tool to rapidly and specifically edit any genomic location. The most direct way of increasing yield is to knock out genes that negatively affect yield (Ma et al., 2016; Song et al., 2016) (Table 1). In one recent case, this was achieved by individually knocking out four negative regulators of yield (the genes *Gn1a*, *DEP1*, *GS3*, and *IPA1*) in the rice cultivar Zhonghua 11 by CRISPR/Cas9. Three of the resulting knockout mutations, *gn1a*, *dep1*, and *gs3*, showed enhanced yield parameters in the T_2_ generation, resulting in improved grain number, dense, erect panicles, and larger grain size, respectively (Li M. et al., 2016). Similarly, Xu et al. (2016) simultaneously knocked out three major rice negative regulators of grain weight (*GW2*, *GW5*, and *TGW6*) using a CRISPR/Cas9-mediated multiplex genome-editing system. The resulting mutants showed a significant increase in thousand-grain weight. Zhang et al. (2016) targeted three homoalleles of *GASR7*, a negative regulator of kernel width and weight in bread wheat, by CRISPR/Cas9 and obtained an increase in the thousand-kernel weight. Similarly, using CRISPR/Cas9 to target a tomato *cis-*regulatory element in the *CLAVATA-WUSCHEL* stem cell circuit (*CLV-WUS*) that controls meristem size produced an edited tomato with an increased number of locules (seed compartments) and thus larger fruit size (Rodríguez-Leal et al., 2017). Moreover, CRISPR/Cas9 has been employed to generate functional knockouts of genes that indirectly contribute to the *improvement* of *yield* characteristics (Lawrenson et al., 2015; Soyk et al., 2016; Braatz et al., 2017; Li and Yang, 2017; Ma et al., 2018; Zhang et al., 2018).

#### Engineering Plant Disease Resistance

Plants are constantly infested by a variety of pathogens, including viruses, bacteria, and fungi (Taylor et al., 2004), that can cause significant losses of crop quality and yield (Savary et al., 2012). Considerable knowledge has been accumulated on the genetic basis of plant disease resistance, and genes related to disease resistance have been identified in different plant species, including Arabidopsis, rice, soybean, potato, tomato, and citrus (Michelmore, 1995; Hammond-Kosack and Jones, 1996).

Genome-engineering technologies have been widely harnessed to engineer plant resistance against pathogens (Ali et al., 2015b; Baltes et al., 2015; Ji et al., 2015; Iqbal et al., 2016) (Table 1). These technologies can be used to target host factors important for pathogen infection and replication, thus immunizing plants against various pathogens. For example, CRISPR/Cas9 was recently used to alter the promoter sequence of the canker susceptibility gene *CsLOB1* in citrus, leading to canker resistance and providing hope for generating disease resistance in citrus varieties (Jia et al., 2016; Peng et al., 2017).

Targeting homologs of *MILDEW-RESISTANCE LOCUS* (*MLO*) and other loci has improved resistance to fungal pathogens in several species. CRISPR/Cas9 and TALEN were successfully used to generate resistance to powdery mildew by simultaneously targeting the three homologs of the *MILDEW-RESISTANCE LOCUS* (*MLO*), *TaMLO-A*, *TaMLO-B*, and *TaMLO-D*, in wheat (Wang et al., 2014). In another example, the Tomelo transgene-free tomato, which is resistant to powdery mildew disease, was developed by targeting the *SlMlo1* gene using CRISPR/Cas9 (Nekrasov et al., 2017). Recently, Zhang et al. (2017) simultaneously modified the three homologs of the wheat *TaEDR1* gene to enhance resistance to powdery mildew disease. In other efforts, knockout of the ethylene-responsive factor (ERF) gene *OsERF922*, a negative regulator of rice blast resistance, enhanced resistance to the blast fungal pathogen (Wang et al., 2016).

Modifications of sucrose transporters have proven successful for resistance against a devastating bacterial pathogen. Using TALENs, Li T. et al. (2012) induced site-specific mutations in the effector binding site of the promoter region of the rice sucrose-efflux transporter gene (*SWEET14*). These mutations affect the survival and virulence of the bacterial leaf blight pathogen *Xanthomonas oryzae* pv. *oryzae* (*Xoo*), resulting in resistant rice lines. CRISPR/Cas9 was also successfully implemented to create mutations in four rice *SWEET* type *S* genes (Zhou et al., 2014). These examples demonstrate the great potential of genome-engineering technologies for producing plant immunity to various pathogens.

##### CRISPR/Cas9-mediated interference against DNA viruses

Plant viruses can have disastrous effects on key staple crops, and the extreme economic impact of some plant virus epidemics and outbreaks is well documented (Legg and Thresh, 2000; Anderson et al., 2004; Sasaya et al., 2014). Genome-engineering technologies can be employed to target viral genomes directly. We and others have recently shown that CRISPR/Cas9 can be harnessed to engineer plant immunity against various DNA geminiviruses, including *Tomato yellow leaf curl virus* (TYLCV), *Beet curly top virus* (BCTV), *Merremia mosaic virus* (MeMV), *Bean yellow dwarf virus* (BeYDV), and *Beet severe curly top virus* (BSCTV) (Ali et al., 2015a, 2016; Baltes et al., 2015; Ji et al., 2015). Interestingly, we found that a single gRNA targeting a conserved region in multiple geminiviruses can mediate interference against multiple viruses, illustrating the great potential of CRISPR/Cas9 as an effective strategy against plant DNA viruses (Ali et al., 2015a).

##### CRISPR/Cas13a-mediated interference against RNA viruses

RNA viruses represent the majority of plant pathogenic viruses, and engineering plant immunity to RNA viruses is increasingly important. We have employed CRISPR/LshCas13a, an RNA-targeting CRISPR/Cas system (Abudayyeh et al., 2016; East-Seletsky et al., 2016; Liu et al., 2017), to engineer interference with an RNA virus, *Turnip mosaic virus* (TuMV), in plants, and thus demonstrated that Cas13a can mediate plant immunity to RNA viruses (Aman et al., 2018). Despite the modest activity of Cas13a against the TuMV-GFP virus, this study highlighted the exciting potential of CRISPR/Cas13 as an antiviral strategy, and it should encourage the identification and development of more robust and effective RNA-targeting CRISPR systems. These will be useful not only for RNA virus interference but also for a variety of RNA targeting and manipulation strategies in plants (Mahas et al., 2017; Ali et al., 2018b).

#### Enhancing Plant Abiotic Stress Tolerance

Abiotic stresses such as drought, salinity, and extreme temperature significantly limit crop yields worldwide by reducing plant growth and development (Pandey et al., 2017). The conditions predicted to result from global climate change will worsen many of these stresses, potentially causing an enormous drop in global crop productivity. Plants withstand various abiotic stresses through elegant response mechanisms that generally involve the expression of multiple stress-inducible genes (Kuzuoglu-Ozturk et al., 2012; Golldack et al., 2014). In particular, transcription factors are keystones in gene regulatory networks that control the expression of many genes involved in stress responses (Singh et al., 2002). Advances in genetics and genomics have improved our understanding of the complex nature of abiotic stresses and the interactions between signaling, regulatory, and metabolic pathway components (Nakashima et al., 2009; Takashi and Kazuo, 2010; Garg et al., 2014; Mickelbart et al., 2015). Numerous potential candidate genes have been identified and transformed by classical genetic engineering methods to improve abiotic stress tolerance in both model plants and agriculturally important crop plants (Bidhan et al., 2011; Gong and Liu, 2013).

Owing to the complex nature of abiotic stress, fewer genome-editing studies have so far been done in this area than in the field of pathogen resistance (Table 1). In one recent study, DuPont scientists successfully modified a gene encoding maize negative regulator of ethylene responses, *ARGOS8*, using CRISPR/Cas9 (Shi et al., 2017). They used the HDR pathway to insert the maize native *GOS2* promoter into the 5′ untranslated region of *ARGOS8*, which resulted in drought-tolerant maize that survives and has better yield under water-deficit conditions. Another group used CRISPR/Cas9 to induce a mutation in the Arabidopsis *OST2* gene; the mutation resulted in an altered stomatal closing pattern in response to environmental conditions, enhancing the plants’ tolerance of drought stress (Osakabe et al., 2016). Recent studies have used CRISPR/Cas9 and validated the involvement of rice *NCED3* and *RAV2* and tomato *MAPK3* in conferring adaptive abiotic stress responses (Duan et al., 2016; Wang et al., 2017; Huang et al., 2018). A recent trial in wheat protoplasts by Kim et al. (2018) targeting two abiotic-stress-responsive transcription factor genes encoding dehydration responsive element binding protein 2 (*TaDREB2*) and ethylene responsive factor 3 (*TaERF3*), further confirmed that CRISPR/Cas9 can be used to manipulate abiotic stress genes for future crop improvement.

#### Enhancing Plant Herbicide Resistance

Weeds compete with crop plants for resources such as water, nutrients, light, and space, causing considerable reductions in yield. Numerous techniques have been used for weed management, especially chemical herbicides and genetic engineering approaches. Herbicides usually target a vital step in a plant metabolic pathway, and therefore completely kill weeds and may cause considerable damage to crop plants as well. The herbicides bring economic benefits by increasing the food supply worldwide, but they can endanger human and animal health and have negative impacts on the environment. The advent of biotechnology has revolutionized farming practices by making it possible to transfer a specific herbicide-resistance gene to multiple crops (Lombardo et al., 2016) (Table 1), allowing the herbicide to selectively kill the weeds without causing damage to the herbicide-tolerant transgenic crops. This approach has greatly reduced the cost of weed control and also somewhat reduced the deleterious effects of these chemicals.

Recently, scientists have begun to use genome editing to knock out endogenous genes, such as *EPSPS* and *ALS*, to produce herbicide-tolerant plants (Lombardo et al., 2016). *ALS* encodes acetolactate synthase, a key enzyme that catalyzes the first step in the biosynthesis of branched-chain amino acids such as valine, leucine, and isoleucine (Lee et al., 1988; Chipman et al., 1998). Its enzymatic activity is inhibited by certain classes of common herbicides, including the sulfonylureas, imidazolinones, triazolopyrimidines, pyrimidinylthio (or oxy) benzoates, and sulfonylamino-carbonyl-triazolinones (Mazur et al., 1987; Zhou et al., 2007). Genome-editing-based gene replacement has been used to introduce precise alterations in the conserved region of *ALS* to prevent its inhibition by these herbicides. The resulting modified plants are able to grow in the presence of herbicide. In 2009, ZFN-mediated gene targeting was first used to introduce specific mutations in the tobacco *ALS* gene to confer resistance to sulfonylurea herbicides (Cai et al., 2009; Shukla et al., 2009; Townsend et al., 2009). The same gene has been targeted in several other crops, using TALENs and CRISPR/Cas9, to obtain herbicide-resistant potato, rice, maize, and soybean varieties (Butler et al., 2015; Svitashev et al., 2015; Li J. et al., 2016; Sun et al., 2016).

*EPSPS* encodes 5-enolpyruvylshikimate-3-phosphate synthase, an enzyme in the shikimate pathway, which is involved in the biosynthesis of essential plant aromatic amino acids (Ganesh and Dilip, 1988). In plants, EPSPS is a target of glyphosate, a widely used herbicide that binds to and inhibits its enzymatic activity (Ganesh and Dilip, 1988; Schönbrunn et al., 2001). CRISPR/Cas9 has been used to substitute two nucleotides in the *EPSPS* glyphosate-binding site in the presence of single-stranded oligo DNA repair templates in *Linum usitatissimum* (flax), resulting in genotypes with elevated glyphosate tolerance (Sauer et al., 2016). A similar approach has been used to produce glyphosate-resistant rice (Li J. et al., 2016).

#### Improving Food Crop Quality

Genome editing can also enhance crop nutritional properties to produce healthier foods. Several studies have proposed potential applications of genome editing in the modification of plant components. For example, phytate, which exists in many crops, is usually regarded as an anti-nutrient due to its ability to form complexes with proteins and minerals, reducing their digestive availability (Zhou and Erdman, 1995; Feil, 2001). TALENs and CRISPR/Cas9 have both been used to reduce phytate content in maize by knocking out *ZmIPK*, a gene involved in phytate biosynthesis (Liang et al., 2014). Another application targeted acrylamide, a potential carcinogen produced by the reaction of reducing sugars (e.g., glucose and fructose) with free amino acids (e.g., in asparagine) in starchy foods, such as potato, under high heat. Clasen et al. (2016) used TALEN to knock out *VInv*, the gene encoding vacuolar invertase, which catalyzes the breakdown of sucrose into glucose and fructose, and thereby produced acrylamide-free potatoes. CRISPR/Cas9 has been used to develop wheat with hypoimmunogenic gluten and tomato with enhanced lycopene content through the generation of functional knockout mutants of α-gliadin genes and several genes involved in carotenoid biosynthesis, respectively (Li et al., 2018; Sánchez-León et al., 2018).

The development of an improved waxy potato is another example of food quality improvement through genome editing. CRISPR/Cas9 was used to knock out the four alleles of the granule-bound starch synthase (*GBSS*) gene in potato. The edited potato produces only amylopectin and lacks amylose-containing starch (Andersson et al., 2017). A similar concept underlies a waxy maize developed by DuPont Pioneer by disrupting the amylose biosynthesis gene (*Wx1*) through CRISPR/Cas9 (Waltz, 2016a). Conversely, a high-amylose rice was generated by knocking out the starch branching enzyme genes *SBEI* and *SBEIIb* using CRISPR/Cas9 (Sun et al., 2017).

Genome editing has also been used to modify seed oil content to produce healthier food oils, as well as biofuels. This approach was made possible by increased knowledge of the metabolic pathways and the genes encoding enzymes related to fatty acid biosynthesis (Wu et al., 2005; Damude and Kinney, 2008). Seed oil content can be modified by increasing and decreasing the levels of particular fatty acids or by incorporating additional fatty acids of nutritional importance. For example, high levels of polyunsaturated fatty acids such as linolenic acid in food oils are undesirable because of their poor oxidative and frying stability. It is now feasible to change fatty acid compositions by targeting the genes encoding fatty acid desaturase (FAD). TALENs have been used to knock out *FAD2-1A* and *FAD2-1B* in soybean, increasing the oleic acid level by almost four times as compared to wild type (Haun et al., 2014). Two independent groups have recently used CRISPR/Cas9 to simultaneously knock out all three *FAD2* homeologs in the allohexaploid oilseed crop *Camelina sativa*, resulting in reduced levels of the less desirable polyunsaturated fatty acids and a significant enhancement of the oleic acid level (Jiang et al., 2017; Morineau et al., 2017).

## Regulation of Genome-Edited Crops

Genome-editing tools have been used to effect precise modifications in many plant genomes. They have had a great influence on basic research as well as crop improvement. A primary advantage of these technologies is that the transgenes initially used to induce genetic alterations can be easily removed from the genome by genetic segregation, making the resulting plants typically indistinguishable from naturally occurring genetic variants. More recent modification methods, especially CRISPR/Cas, have improved the robustness of this process by allowing genetic changes to be accomplished without any integration of foreign DNA, through transient expression of a site-specific nuclease within the plant cell (Weeks et al., 2016). The transient nature of the expression often results from the degradation of nuclease-encoding DNA constructs after they have done their job and before they can be integrated into the plant’s genome. This can be achieved by using viral vectors to deliver the site-specific nuclease in the form of either mRNA, which is unstable and quickly degrades, or protein, which is not transmitted from parent to offspring (Marton et al., 2010; Baltes et al., 2014; Ali et al., 2015b; Ilardi and Tavazza, 2015; Yin et al., 2015). In these cases, we argue that the edited plants should not be regulated in the same way as those generated by classical genetic engineering methods (Sauer et al., 2016).

Scientists, policymakers, and regulatory authorities have extensively debated the regulation of genome-edited plants (European Food Safety Authority Panel on Genetically Modified Organisms, 2012; Lusser and Davies, 2013; Podevin et al., 2013; Pauwels et al., 2014). Among the numerous issues discussed are such questions as whether genome-edited plants should be regulated under the existing frameworks for GMOs. Should regulations consider process-based regulation, which considers the procedures and techniques used to create the crop, or product-based regulation, which considers the possible risk of the final crop products? Should they deal with edited plants on a case-by-case basis according to parameters such as (1) the tool and repair pathway employed (NHEJ versus HDR), (2) the characteristics of the developed or modified trait, and (3) the possibility of off-target effects (Araki et al., 2014; Hartung and Schiemann, 2014; Araki and Ishii, 2015; Wolt et al., 2016)?

The United States Department of Agriculture (USDA) stated in 2012 that plants edited with ZFNs and meganucleases using the NHEJ pathway should not be considered as, or regulated as, GMOs (Waltz, 2012). The USDA has followed this product-based distinction in later judgments and recently allowed the cultivation and commercialization of CRISPR-edited mushrooms and waxy corn without passing them through the existing GMO regulation (Waltz, 2016b). DuPont Pioneer is planning to release the waxy corn variety as the first commercialized genome-edited crop in 2020. The European Union (EU) regulations are mainly process based. Nonetheless, various anti-GMO forces consider genome-edited plants to be unnatural products and are attempting to have them banned under the GMO regulatory scheme. These arguments are illogical, however, given that the EU previously approved several older crops created by the even more imprecise conventional methods of chemical and radiation mutagenesis. Very recently, however, a ruling by the European Court of Justice (ECJ) included CRISPR-edited crops within the GMO category, complicating commercialization efforts and severely undercutting CRISPR-based efforts for crop trait improvements in Europe and other markets with intensive agricultural trade relations with European countries (Urnov et al., 2018). We certainly hope that this decision will be revisited and that a science-based and informed decision is made on this matter. This decision should take into consideration the opportunities to use this technology to address agricultural challenges and enhance food security globally (Urnov et al., 2018).

In practical terms, genome-editing technologies offer a great chance for improving crops and ensuring global food security. We should grasp this opportunity to increase crop productivity and potentially save the lives of millions of people around the world, particularly in developing nations. Treating genome-edited crops like those produced naturally or by older artificial mutagenesis will have a number of positive impacts on global food security, including (1) reducing the time and cost of regulatory scrutiny, which will encourage more small biotechnology companies to adopt genome editing; (2) increasing the number of researchers using these tools and encouraging them to improve the system’s efficiency and develop more robust techniques; and (3) allowing the technology to be applied to more crops, including food and horticultural species. As a result, revolutionary changes in crop improvement can be expected in the near future to help meet the increasing demand for food and ensure global food security.

## Conclusion

CRISPR/Cas systems have revolutionized plant genome engineering and democratized their application through their high efficiency, facile engineering, and robustness. The current state of this technology enables many applications suitable for improving plant productivity, disease resistance, and resilience to climate change. Various technological improvements are still needed, especially precise editing and delivery of genome-engineering reagents to germline cells to bypass the need for tissue culture. In addition, regulatory and ethical considerations may limit the wide applications of these technologies. We must learn from past experience and improve the technology to avoid regulatory hurdles and ensure that its fruits are within reach for the poor and for subsistence farmers. Genome-editing technologies are poised to reshape the future of plant agriculture and food security to feed the world’s burgeoning population.

## Author Contributions

KS, AM, and MM wrote the manuscript. KS, AM, and MM prepared the figures and table. KS and MM edited and finalized the manuscript.

## Conflict of Interest Statement

The authors declare that the research was conducted in the absence of any commercial or financial relationships that could be construed as a potential conflict of interest.
